# Health-adjusted life expectancy according to lifestyle classified by the Yonsei Lifestyle Profile-BREF

**DOI:** 10.4178/epih.e2022095

**Published:** 2022-10-28

**Authors:** Sanghun Nam, Kang-Hyun Park, Ji-Hyuk Park, Ickpyo Hong

**Affiliations:** 1 Department of Occupational Therapy, Graduate School, Yonsei University, Wonju, Korea; 2 Department of Occupational Therapy, Baekseok University, Cheonan, Korea; 3 Department of Occupational Therapy, College of Software and Digital Healthcare Convergence, Yonsei University, Wonju, Korea

**Keywords:** Lifestyle, Life expectancy, Aged

## Abstract

**OBJECTIVES:**

This study aimed to investigate health-adjusted life expectancy (HALE) by demographic characteristics (sex, educational achievement, and residential area) according to the lifestyle classifications of the Yonsei Lifestyle Profile-BREF (YLP-BREF).

**METHODS:**

This study included 569 participants aged 55 years or older living in Korea. The YLP-BREF domains were physical activity, activity participation, and nutrition.

**RESULTS:**

Females had a longer HALE (mean±standard deviation, 8.90±5.06 years) in the physical activity domain, while males had a longer HALE in the nutrition domain (9.44±5.91 years). People living in rural areas had longer HALE in physical activity (12.02±5.60 years), activity participation (8.58±4.21 years), and nutrition (11.33±6.43 years). There were no significant differences according to sex or residential area. High school graduates showed the longest HALE (physical activity: 10.38± 6.89; activity participation: 7.64±4.29; nutrition: 9.59±6.40 years). There was a significant difference in educational achievement.

**CONCLUSIONS:**

As people age, the demand for a healthy lifestyle increases. This study attempted to calculate HALE by demographic characteristics according to lifestyle. The results of this study will help inform future research directions for providing a healthy lifestyle.

## INTRODUCTION

Population aging is a global phenomenon [1]. Every country in the world is experiencing an increase in the proportion of older adults in their population [1]. Furthermore, older adults are living longer; thus, there is a trend for improved survival in individuals aged 65 and above worldwide [1,2]. With the shift of demographic structures toward an aging population, significant pressure is placed on the working-age population [3]. Therefore, the health and functional ability of older adults are vital in determining the potential productivity and healthcare costs of the aging population [4]. However, population aging potentially leads to increased medical costs and long-term care services for older adults in the population [5]. With an increasing dependency ratio of older adults, it is expected that older people will become more dependent on social services and that healthcare needs will increase due to the conditions that accompany old age [6].

Under these circumstances, interest is increasingly directed more toward living a healthy life than simply living longer. In the past, life expectancy (LE) was an important indicator of population health status [7]. However, research has found that LE is insensitive to the health status of the population, as it cannot be used as an indicator of the quality of life, only the quantity [8,9]. Moreover, LE presents a critical weakness as it cannot reflect morbidity, disability, or health status [10]. To compensate for the disadvantages of LE [9,10], the concept of health-adjusted life expectancy (HALE) was developed in 1972 [11]. HALE is a summary measure that can estimate the average number of years that a person at a given age is expected to live in a completely healthy state [12]. The World Health Organization (WHO) used HALE as an official measurement method for annual reports to provide information on the average level of health of the population of member states [13]. Therefore, it is critical to consider not only the length of life, but also the HALE of older adults in order to increase the population’s health and well-being.

A healthy lifestyle is considered to be an important factor for improving the HALE of older adults population [14]. The WHO stated that 60% of the factors affecting individuals’ health are lifestyle-related characteristics. An unhealthy or unbalanced lifestyle causes various diseases, such as metabolic diseases, hypertension, joint and skeletal problems, and cardiovascular diseases [15,16]. Since lifestyle is associated with health and mortality, it is important to determine whether older people have healthier lifestyles.

Several lifestyle assessment tools exist, including FANTASTIC [17], the Health Enhancement Lifestyle Profile [18], and the Personal Lifestyle Questionnaire [19]. Although all these evaluation tools have been verified, there are some limitations to their application to older adults [20]. Specifically, existing evaluation tools do not focus on elderly individuals [21]. These measures also tend to focus on assessing the frequency and duration of specific lifestyle factors such as exercise, diet, smoking, and drinking [22-26]. Furthermore, most lifestyle evaluation tools were developed based on Western cultures. Therefore, these lifestyle assessments cannot measure the lifestyle of older adults in Asian cultures, as lifestyles tend to be shaped by the environment [27].

The Yonsei Lifestyle Profile-BREF (YLP-BREF) was developed to examine physical activity, activity participation, and nutrition and to identify individuals who engage in a healthy lifestyle [28]. The YLP-BREF includes 22 items related to 3 life elements: physical activity, activity participation, and nutrition. There are 5 physical activity questions, 4 activity participation questions, and 13 nutrition questions (a total of 22 questions). All 22 items are rated on a 5-point Likert scale. Participants respond by indicating the frequency with which they engage in a particular activity. Since the YLP-BREF has cut-off scores for each domain, it can classify individuals who have a healthy lifestyle.

Hence, it is worthwhile to analyze HALE by demographic characteristics according to lifestyle factors. Therefore, this study aimed to examine HALE by demographic characteristics (sex, educational achievement, and residential area) according to the lifestyle classifications of the YLP-BREF.

This study calculated HALE by dividing participants into those with a healthy lifestyle (1) and unhealthy lifestyle (0) using the YLP-BERF. After generating the classification of 0 or 1 using the cut-off score of YLP-BREF, the proportion of all participants with a score of 1 was calculated and applied as a weight when calculating HALE. In addition, HALE was divided into 3 variables, corresponding to different aspects of lifestyle, based on previous studies finding that there were differences in lifestyle according to demographic characteristics.

## MATERIALS AND METHODS

### Data source and study design

The data used in this study were obtained from a study by Park et al. [29]. The subjects included in this dataset were 569 people 55 years of age or older residing in Korea between September and November 2020. The following were the criteria for collecting data: (1) people aged 55 years or older living in the local community and (2) people who have lived in Korea in recent years.

### The Yonsei Lifestyle Profile-BREF

The original Yonsei Lifestyle Profile (YLP) is a 60-item questionnaire designed to measure the lifestyle of an individual in 3 domains—namely, physical activity, activity participation, and nutrition [30,31]. The YLP is composed of a 5-point Likert scale. The physical activity and activity participation domains consist of items on frequency, time, and satisfaction, and the nutrition domain consists of items on frequency and satisfaction. The YLP exhibited high internal reliability, with a Cronbach’s alpha of 0.83. The test-retest reliability of YLP was 0.97 [31]. In contrast, the YLP-BREF consists of 22 items from the 60-item YLP [28]. The YLP-BREF consists of only frequency items. For example, physical activity and activity participation are assessed with items that have the following responses: (1) never, (2) 1-2 day/wk, (3) 3-4 day/wk, (4) 5-6 day/wk, and (5) 7 day/wk. The items on nutrition have the following responses: (1) never, (2) 1-2 day/wk, (3) 3-4 day/wk, (4) 5-6 day/wk, and (5) 7 day/wk. There are 5 frequency items in the physical activity domain, 4 in the activity participation domain, and 13 in the nutrition domain. A previous study reported that the cut-off scores for each domain of the YLP-BREF were 9 points for physical activity, 6 points for activity participation, and 28 points for nutrition [28]. In this study, using the cut-off scores of YLPBREF, HALE was according to whether individuals had a healthy lifestyle (defined as a score of 1) or an unhealthy lifestyle (defined as a score of 0).

### Defining health-adjusted life expectancy

The term “HALE” has recently been used to describe the same concept as disability-adjusted LE [32]. HALE is an estimate of the average number of years that a person at a given age is expected to live in a healthy state [12]. The “health” in HALE is selected using panel data or sample cohort’s data on health-related quality of life or illness in a population. “Health” is usually expressed as a degree of disability [33]. The degree of disability is assessed using disability weights, which quantify the level of disability associated with a particular health condition or disease on a scale from 1 (completely healthy) to 0 (having a disability) [34]. Thus, compared to disease-free or disability-free LE, which evaluates the disability level as 0 or 1, HALE can more accurately account for disability levels. We calculated HALE using the cut-off score of YLP-BREF as the disability weight in the existing HALE calculation method. Therefore, in this study, disability weights were calculated based on a division into 0 (unhealthy lifestyle) and 1 (healthy lifestyle); specifically, the disability weight was the proportion of the total number of participants who had a healthy lifestyle. Since the YLP data used in this study were collected in 2020, the 2020 data from the life table related to mortality were utilized [35]. HALE was measured as follows: (1) The number of years of survival by age (*L_x_*) was extracted from the life table data provided by Statistics Korea. (2) The prevalence of healthy lifestyles (*Wt_x_*) for each YLP domain was calculated (Supplementary Materials 1-3). The prevalence of healthy lifestyles was calculated as the proportion of participants with a certain value for an independent variable (demographic characteristics: sex, educational attainment, region) who had healthy lifestyles. (3) The number of years of survival by age was multiplied by the prevalence of each YLP domain to obtain the weight (*L'_x_*) of the number of years of survival by age. (4) HALE (*e'_x_*) was obtained by dividing the total number of years of survival (*T_x_*), which is the total number of years of survival at each age, by the number of survivors by age (*I_x_*). HALE can be measured using the following formulas:


(1) $$L{'}_{x}=L_{x}\times Wt_{x}$$

(2) $$T_{x}=\sum L{'}_{x}$$

(3) $$e{'}_{x}=T_{x}/I_{x}$$

### Statistical analysis

A descriptive analysis was conducted to investigate demographic characteristics. The HALE according to the YLP-BREF was calculated using the Sullivan method. SAS version 9.4 (SAS Institute Inc., Cary, NC, USA) was used for data management and data analysis. We used sex, educational achievement, and residential area variables to calculate HALE for each demographic characteristic according to lifestyle.

### Ethics statement

This study was approved by the Yonsei University Institutional Review Board (#1041849-202111-SB-202-01) and met the research exemption criteria of all participating institutions.

## RESULTS

Table 1 presents the demographic characteristics of participants who had complete YLP-BREF responses. This study included a total of 569 participants, with an average age of 60.23±4.26 years. Slightly more than half of the participants (n= 287, 50.44%) were males. Furthermore, 395 (69.42%) of the participants had a college degree, and 13 (2.28%) had an educational status of only middle school graduation or less. Regarding employment status, 296 (52.02%) of the participants were working, and 12 (2.11%) were not. The number of people with chronic diseases was 291 (51.14%), corresponding to more than half the total number of participants. Lastly, 304 (53.43%) of the participants lived in urban areas and 10 (1.76%) lived in rural areas.

### Yonsei Lifestyle Profile-BREF health-adjusted life expectancy by sex

Table 2 presents the HALE for each YLP-BREF domain according to sex. The LE according to the life table of Statistics Korea was 21.38± 3.95 years on average for males aged 55-70 years and 25.93± 4.40 years for females aged 55-70 years. For the physical activity domain, the HALE of males was 8.62± 4.99 years, whereas that of females was 8.90± 5.06 years. For the activity participation domain, the HALE of males were 6.89± 7.91 years, whereas that of females were 7.91± 3.71 years. For the nutrition domain, the HALE of males were 9.44± 5.91 years, whereas that of females were 8.63± 4.70 years.

### Yonsei Lifestyle Profile-BREF health-adjusted life expectancy by educational attainment

Table 2 presents the HALE for each YLP-BREF domain according to educational achievement. According to the life table of Statistics Korea, the LE of middle school graduates or less between the ages of 55 and 70 was 24.40± 3.49. For high school graduates aged 55 to 70 years, the LE was 23.72± 4.15, whereas for college graduates, it was 23.72± 4.15 years. For the physical activity domain, middle school graduates or less had the shortest HALE (2.97± 1.33 years), whereas high school graduates had the longest HALE (10.38± 6.89 years). For the activity participation domain, middle school graduates or less had the shortest HALE (3.29± 1.53 years), whereas high school graduates had the longest HALE (7.64± 4.29 years). For the nutrition domain, middle school graduates or less had the shortest HALE (3.34± 1.51 years), whereas high school graduates had the longest HALE (9.59± 6.40 years).

### Yonsei Lifestyle Profile-BREF health-adjusted life expectancy by residential area

Table 2 presents the HALE for each YLP-BREF domain according to the residential area. According to the life table of Statistics Korea, the LE of urban residents aged 55 to 70 was 23.72± 4.15 years, whereas that of suburban residents aged 55 to 70 was 23.72± 4.15. The LE of rural residents between the ages of 55 and 70 was 25.36± 4.62 years. For the physical activity domain, rural residents had the longest HALE (12.02± 5.60 years), whereas urban residents had the shortest HALE (8.44± 4.69 years). For the activity participation domain, rural dwellers had the longest HALE (8.58± 4.21 years), whereas urban residents had the shortest HALE (7.21± 3.81 years). For the nutrition domain, rural residents had the longest HALE (11.33± 6.43 years), whereas urban residents had the shortest HALE (9.11± 5.25 years).

## DISCUSSION

This study aimed to determine whether there were differences in HALE between demographic characteristics according to lifestyle. In this study, healthy and unhealthy lifestyles were classified for each domain of the YLP-BREF. Using the weights of the classified lifestyles, HALE was calculated by demographic characteristics according to physical activity, activity participation, and nutrition. Differences in HALE appeared only according to educational achievement, and there was no significant difference according to sex or residential area.

HALE, according to sex, was longer in the physical activity domain for females and in the nutrition domain for males, but there was no significant difference. In a previous study, data from the Korea National Health and Nutrition Examination Survey were used to analyze differences in dietary habits and nutritional status between single-person and multi-person households. It was reported that, as age increased, the level of nutritional intake decreased in single-person female households more than in singleperson male households [36]. Furthermore, although the physique and physical function of older Korean females decreased with increasing age, no significant decrease in physical activity was observed [37]. In contrast, among males in their 20s to 60s, as their age increases, their muscle mass decreases, and as their body fat increases, their physical activity level also decreases [38]. Correspondingly, activity participation was also higher among females than males. Previous studies showed differences in physical activity and activity participation according to sex, but in our study, no significant differences were found between males and females. Despite the lack of statistical significance, the numerical difference in HALE showed the same direction as in the previous study.

The HALE by educational achievement according to each YLP-BREF domain was, on average, longest among high school graduates and shortest among middle school graduates or less, showing a significant difference. This indicates that physical activity decreases as age increases and educational attainment decreases [39,40]. As such, physical activity is related to educational achievement [41]. Accordingly, it can be observed that HALE according to the physical activity domain increases as educational achievement increases. In addition, Hall [42] reported that higher educational attainment increased the proportion of participation in social and leisure activities. In other words, as activity participation increases with higher levels of educational achievement, HALE in activity participation domain increases as the level of educational achievement becomes higher. Lastly, higher educational achievement was associated with a greater increase in HALE in the nutrition domain. Many studies have reported income inequalities according to educational attainment [43,44]. Higher educational attainment is associated with higher income and, accordingly, differences in nutritional intake. In addition, a study by Cembranel et al. [45] reported a higher intake of micronutrients among people with higher educational attainment than among those having lower educational attainment. Accordingly, higher educational achievement is associated with longer HALE in the nutrition domain.

In the area of physical activity, the HALE value was higher in rural areas than in urban areas, but without a significant difference. According to previous research, individuals living in urban areas are less physically active than individuals in rural areas [46,47]. Furthermore, individuals living in urban areas engage in less physical activity due to the convenience of available facilities, whereas individuals in rural areas engage in more physical activity due to the rural environment. According to a study by Lee et al. [48], rural residents reported more social and organizational participation, such as trust in neighbors, exchange of help with neighbors, and socializing with friends. Finally, in the nutritional domain, the HALE value was higher in rural areas than in urban areas, but again without a significant difference. Chung & Kim [49] reported that older adults living in urban areas consumed more meat, fish, milk, oily foods, ice cream, cakes, snacks, and carbonated drinks, whereas those in rural areas consumed more vegetables than urban dwellers. Accordingly, it has been reported that metabolic syndrome, the components of which include hyperlipidemia and hypertension, is highly likely to develop in older adults living in urban areas. Unlike previous studies, our study showed no significant difference in HALE values between residential areas. However, the direction of the HALE values showed similar results to those of previous studies. One of the reasons why no significant differences were found in our study may have been the impact of rural development. Kim [50] reported that the economic disparity between urban and rural areas was alleviated due to the revitalization of tourist destinations in rural areas. Reducing the financial gap leads to an increase in the quality of life in rural areas, which may explain the lack of a significant difference in this study, unlike previous studies.

The purpose of this study was to calculate HALE according to demographic characteristics using the YLP-BREF. According to each calculated YLP-BREF domain, HALE varied according to demographic characteristics. However, significant differences were not found in a comparative statistical analysis of HALE according to sex and residential area (Supplementary Materials 4-6). As discussed above, previous studies have described reasons why lifestyles vary according to sex and residential area. The absence of statistically significant differences in HALE according to sex and residential area in our study seems to have been due to the biased data of 556 patients. Therefore, future studies should perform statistical comparisons by collecting various samples and applying the methodology of this study.

This study has several limitations. First, the data used were from people aged 55 years or older living in Korea. Therefore, the results cannot be generalized to adolescents and young adults. That is, the results may have been influenced by the age of the participants in the YLP-BREF survey. Second, since the YLP-BREF is a self-report questionnaire, it is affected by the tendency to provide socially desirable answers. Finally, this study used data from 569 participants that had already been collected. Despite using data from 569 participants, the number of participants with middle school education or less was relatively small. The LE calculated by educational attainment for middle school graduates or less was based on a few people, and the results might therefore not be generalizable. Therefore, it is necessary to analyze data from participants with various levels of educational attainment in future research.

Our study calculated HALE according to demographic characteristics and lifestyle. For each domain of the YLP-BREF, no significant differences were found in HALE by sex or region, whereas there was a significant difference in HALE by education. The results of this study may help in research on interventions for healthy lifestyles and related health programs in the future. However, since data from a small sample were used in this study, it is necessary to calculate HALE according to lifestyle data from a broader variety of older adults in future studies.

## Figures and Tables

**Table 1.** Demographic characteristics of respondents to the YLP-BREF (n=569)

Characteristics	n (%)
Age, mean±SD (yr)	60.23±4.26
Sex	
Male	287 (50.44)
Female	282 (49.56)
Employment status	
Employed	296 (52.02)
On temporary leave	53 (9.31)
Resigned	12 (2.11)
Retired	87 (15.29)
Full-time housewife	121 (21.27)
Educational attainment	
Middle school graduation or less	13 (2.28)
High school	161 (28.30)
College degree	395 (69.42)
Chronic conditions (yes)	291 (51.14)
Residential area	
Urban	304 (53.43)
Suburban	255 (44.82)
Rural	10 (1.76)

YLP-BREF, Yonsei Lifestyle Profile-BREF; SD, standard deviation.

**Table 2.** YLP-BREF health-adjusted life expectancy by sex, educational attainment, residential area

Variables	Sex		Educational attainment			Residential area		
	Male	Female	Middle school graduation or less	High school	College degree	Urban	Suburban	Rural
Age (yr)	55-70	55-70	55-70	55-70	55-70	55-70	55-70	55-70
LE (total)	21.38±3.95	25.93±4.40	24.40±3.49	23.72±4.15	23.72±4.15	23.72±4.15	23.72±4.15	25.36±4.62
Physical activity	8.62±4.99	8.90±5.06	2.97±1.33	10.38±6.89	8.16±4.60	8.44±4.69	9.24±5.69	12.02±5.60
Participation in activities	6.89±3.70	7.91±3.71	3.29±1.53	7.64±4.29	7.06±3.53	7.21±3.81	7.73±3.79	8.58±4.21
Nutrition	9.44±5.91	8.63±4.70	3.34±1.51	9.59±6.40	8.92±5.38	9.11±5.25	9.21±5.90	11.33±6.43

Values are presented as mean±standard deviation. YLP-BREF, Yonsei Lifestyle Profile-BREF; HALE, health-adjusted life expectancy; LE, life expectancy.

## SUPPLEMENTARY MATERIALS

Supplementary Material 1. Sex weighting according to YLP-BREF

Supplementary Material 2. Educational achievement weight according to YLP-BREF

Supplementary Material 3. Residential area weights according to YLP-BREF

Supplementary Material 4. t-test analysis of health-adjusted life expectancy with sex

Supplementary Material 5. ANOVA analysis of health-adjusted life expectancy with education attainment

Supplementary Material 6. ANOVA analysis of health-adjusted life expectancy with residential area

## References

[1] United Nations World population prospects 2019 [cited 2022 Apr 1]. https://www.un.org/development/desa/pd/news/world-population-prospects-2019-0.

[2] Kramarow E, Lubitz J, Lentzner H, Gorina Y (2007). Trends in the health of older Americans, 1970-2005. Health Aff (Millwood).

[3] Andor L (2012). Employment trends and policies for older workers in the recession. https://www.eurofound.europa.eu/sites/default/files/ef_publication/field_ef_document/ef1235en.pdf.

[4] Mehta N, Myrskylä M (2017). The population health benefits of a healthy lifestyle: life expectancy increased and onset of disability delayed. Health Aff (Millwood).

[5] Cristea M, Noja GG, Stefea P, Sala AL (2020). The impact of population aging and public health support on EU labor markets. Int J Environ Res Public Health.

[6] Gray A (2005). Population ageing and health care expenditure. Ageing Horiz.

[7] National Library of Medicine MeSH subject headings 2022 [cited 2022 Feb 15]. http://www.ncbi.nlm.nih.gov/mesh/?term=life+expectancy.

[8] Wolfson MC (1996). Health-adjusted life expectancy. Health Rep.

[9] Field MJ, Gold MR (1998). Summarizing population health: directions for the development and application of population metrics.

[10] Murray CJ, Salomon JA, Mathers CD, Lopez AD, World Health Organization (2002). Summary measures of population health: concepts, ethics, measurement and applications. https://apps.who.int/iris/handle/10665/42439.

[11] Sullivan DF (1971). A single index of mortality and morbidity. HSMHA Health Rep.

[12] Gold MR, Stevenson D, Fryback DG (2002). HALYS and QALYS and DALYS, Oh My: similarities and differences in summary measures of population health. Annu Rev Public Health.

[13] Farhud DD (2015). Impact of lifestyle on health. Iran J Public Health.

[14] Hu FB, Liu Y, Willett WC (2011). Preventing chronic diseases by promoting healthy diet and lifestyle: public policy implications for China. Obes Rev.

[15] Reddy P, Rankins D, Timoshanko A, Dunbar JA (2011). Life! in Australia: translating prevention research into a large-scale intervention. Br J Diabetes Vasc Dis.

[16] Pahor M, Guralnik JM, Ambrosius WT, Blair S, Bonds DE, Church TS (2014). Effect of structured physical activity on prevention of major mobility disability in older adults: the LIFE study randomized clinical trial. JAMA.

[17] Wilson DM, Ciliska D (1984). Lifestyle assessment. Can Fam Physician.

[18] Hwang JE (2010). Reliability and validity of the health enhancement lifestyle profile (HELP). OTJR (Thorofare N J).

[19] Mahon NE, Yarcheski A, Yarcheski TJ (2002). Psychometric evaluation of the personal lifestyle questionnaire for adolescents. Res Nurs Health.

[20] Park KH, Won KA, Park JH (2019). A systematic study on the multifaceted lifestyle assessment tools for community-dwelling elderly: trend and application prospect. Ther Sci Rehabil.

[21] Yang J, Choi M (2005). A study on leisure participation and satisfaction according to the lifestyle of the elderly. J Sport Leis Stud.

[22] Aygar H, Zencirci SA, Emiral GO, Alaiye M, Soysal A, Onsuz MF (2019). Assessment of health-promoting lifestyle behaviors of adults living in the semi-rural area. North Clin Istanb.

[23] Elley CR, Dawes D, Dawes M, Price M, Draper H, Goodyear-Smith F (2014). Screening for lifestyle and mental health risk factors in the waiting room: feasibility study of the Case-finding Health Assessment Tool. Can Fam Physician.

[24] Hwang JE (2012). Development and validation of a 15-item lifestyle screening for community-dwelling older adults. Am J Occup Ther.

[25] Sutherland LL, Weiler DM, Bond L, Simonson S, Reis J (2012). Northwest Latinos’ health promotion lifestyle profiles according to diabetes risk status. J Immigr Minor Health.

[26] Tambağ H, Öz F (2013). Evaluation of the psychoeducation given to the elderly at nursing homes for a healthy lifestyle and developing life satisfaction. Community Ment Health J.

[27] Moschis GP (1987). Consumer socialization: a life-cycle perspective.

[28] Park KH, Nam S, Hong I, Park JH (2022). An investigation of the psychometric properties of lifestyle profile-BREF. Phys Occup Ther Geriatr.

[29] Park KH, Yoo EY, Kim J, Hong I, Lee JS, Park JH (2021). Applying latent profile analysis to identify lifestyle profiles and their association with loneliness and quality of life among community-dwelling middle- and older-aged adults in South Korea. Int J Environ Res Public Health.

[30] Park KH, Park JH (2020). Development of an elderly lifestyle profile: a Delphi survey of multidisciplinary health-care experts. PLoS One.

[31] Park JH, Park KH, Han DS (2021). The Yonsei Lifestyle Profile for adults and the older adults: development and test‐retest reliability. Alzheimers Dement.

[32] GBD 2017 DALYs and HALE Collaborators (2018). Global, regional, and national disability-adjusted life-years (DALYs) for 359 diseases and injuries and healthy life expectancy (HALE) for 195 countries and territories, 1990-2017: a systematic analysis for the Global Burden of Disease Study 2017. Lancet.

[33] Manuel DG, Schultz SE, Kopec JA (2002). Measuring the health burden of chronic disease and injury using health adjusted life expectancy and the Health Utilities Index. J Epidemiol Community Health.

[34] Ock M, Ko S, Lee HJ, Jo MW (2016). Review of issues for disability weight studies. Health Policy Manag.

[35] Statistics Korea Statistics Korea life table released in 2020 2021 [cited 2022 Jan 17]. http://manibaesang.co.kr/bbs/board.php?bo_table=notice&wr_id=4.

[36] Ahn Y, Lee Y, Park H, Song K (2021). Gender and age group differences in nutrition intake and dietary quality of Korean adults eating alone: based on Korean National Health and Nutrition Examination Survey Data, 2013-2016. Nutr Res Pract.

[37] Kong S (2019). Comparison of physique, physical functions and physical activity by age among Korean elderly women. J Coach Dev.

[38] Shin YA, Ok J (2012). Relationships between age, physical activity and obesity index in men. J Wellness.

[39] Shaw BA, Spokane LS (2008). Examining the association between education level and physical activity changes during early old age. J Aging Health.

[40] Kaplan MS, Newsom JT, McFarland BH, Lu L (2001). Demographic and psychosocial correlates of physical activity in late life. Am J Prev Med.

[41] Saito Y, Oguma Y, Inoue S, Tanaka A, Kobori Y (2013). Environmental and individual correlates of various types of physical activity among community-dwelling middle-aged and elderly Japanese. Int J Environ Res Public Health.

[42] Hall JD (2018). The effects of the quality and quantity of education on income inequality. Econ Bull.

[43] Panori A, Psycharis Y (2019). Exploring the links between education and income inequality at the municipal level in Greece. Appl Spat Anal Policy.

[44] Arshed N, Anwar A, Hassan MS, Bukhari S (2019). Education stock and its implication for income inequality: the case of Asian economies. Rev Dev Econ.

[45] Cembranel F, Wagner KJ, González-Chica DA, d’Orsi E (2020). Education and income levels are associated with energy and micronutrient intake. Int J Vitam Nutr Res.

[46] Jee Y, Goo C (2007). The differences of abilities to physical activity, social networks, depression degrees of elderly between residing rural and urban. J Coach Dev.

[47] Oh SH, Yoon DS (2006). A comparative study on quality of life on the elderly among urban, farm and island area. J Welf Aged.

[48] Lee JA, Park JH, Kim M (2015). Social and physical environments and self-rated health in urban and rural communities in Korea. Int J Environ Res Public Health.

[49] Chung E, Kim M (2020). Correlates of sedentary behavior in Korean older adults: a cross-sectional comparison of elders in urban and rural areas. J Korean Gerontol Nurs.

[50] Kim JH (2021). The effect of rural tourism on mitigating economic disparity between urban and rural areas. J Hosp Tour Stud.

