# Supplementary material for: Health-adjusted life expectancy according to lifestyle classified by the Yonsei Lifestyle Profile-BREF
Source: Epidemiol Health. 2022 Oct 28;44:e2022095. doi: 10.4178/epih.e2022095 (PMC10396514; doi:10.4178/epih.e2022095)
Supplement: Supplementary Material 1. — Sex weighting according to YLP-BREF [file epih-44-e2022095-Supplementary-1.docx]

Supplementary Material 1. Sex weighting according to YLP-BREF

| Age | Male | Female |
| --- | --- | --- |
| 55 | 0.9 | 0.8788 |
| 56 | 1 | 0.8571 |
| 57 | 0.9355 | 0.7273 |
| 58 | 0.9643 | 0.8462 |
| 59 | 0.8214 | 0.9 |
| 60 | 0.9333 | 0.7308 |
| 61 | 0.84 | 0.7826 |
| 62 | 0.7917 | 0.6154 |
| 63 | 0.9 | 0.7727 |
| 64 | 1 | 0.5 |
| 65 | 0.9091 | 0.7778 |
| 66 | 0.375 | 0.8 |
| 67 | 0.4286 | 0.5 |
| 68 | 0.75 | 0.4 |
| 69 | 0.6667 | 0.8333 |
| 70 | 0.375 | 1 |
