# Supplementary material for: Health-adjusted life expectancy according to lifestyle classified by the Yonsei Lifestyle Profile-BREF
Source: Epidemiol Health. 2022 Oct 28;44:e2022095. doi: 10.4178/epih.e2022095 (PMC10396514; doi:10.4178/epih.e2022095)
Supplement: Supplementary Material 2. — Educational achievement weight according to YLP-BREF [file epih-44-e2022095-Supplementary-2.docx]

Supplementary Material 2. Educational achievement weight according to YLP-BREF

| Age | Middle school or less | High school | College degree |
| --- | --- | --- | --- |
| 55 | 1 | 0.8 | 0.9286 |
| 56 | . | 0.8 | 0.9565 |
| 57 | . | 0.7857 | 0.8718 |
| 58 | . | 0.875 | 0.9211 |
| 59 | 1 | 0.6923 | 0.9118 |
| 60 | . | 1 | 0.8085 |
| 61 | 1 | 1 | 0.7805 |
| 62 | 1 | 0.8125 | 0.625 |
| 63 | 0.5 | 0.9091 | 0.7895 |
| 64 | . | 0.625 | 0.7778 |
| 65 | 1 | 0.7143 | 0.9 |
| 66 | . | 0.6 | 0.5 |
| 67 | 0 | 0.6 | 0.4286 |
| 68 | . | 0.5 | 0.6 |
| 69 | . | 1 | 0.75 |
| 70 | . | 0.5 | 0.5 |
