# Supplementary material for: Health-adjusted life expectancy according to lifestyle classified by the Yonsei Lifestyle Profile-BREF
Source: Epidemiol Health. 2022 Oct 28;44:e2022095. doi: 10.4178/epih.e2022095 (PMC10396514; doi:10.4178/epih.e2022095)
Supplement: Supplementary Material 3. — Residential area weights according to YLP-BREF [file epih-44-e2022095-Supplementary-3.docx]

Supplementary Material 3. Residential area weights according to YLP-BREF

| Age | Urban | Suburban | Rural |
| --- | --- | --- | --- |
| 55 | 0.9302 | 0.7895 | 1 |
| 56 | 0.9444 | 1 | 0 |
| 57 | 0.8286 | 0.8824 | 1 |
| 58 | 0.907 | 0.9 | 1 |
| 59 | 0.8611 | 0.8333 | . |
| 60 | 0.7949 | 0.9375 | 1 |
| 61 | 0.8421 | 0.7 | . |
| 62 | 0.6316 | 0.9167 | . |
| 63 | 0.8095 | 1 | 0 |
| 64 | 0.7692 | 0.5 | . |
| 65 | 0.8571 | 0.8333 | . |
| 66 | 0.6667 | 0.3333 | 0 |
| 67 | 0.625 | 0.2 | . |
| 68 | 0.6 | 0.5 | . |
| 69 | 0.7143 | 1 | . |
| 70 | 0.5 | 0.6667 | 0 |
