# Supplementary material for: Health-adjusted life expectancy according to lifestyle classified by the Yonsei Lifestyle Profile-BREF
Source: Epidemiol Health. 2022 Oct 28;44:e2022095. doi: 10.4178/epih.e2022095 (PMC10396514; doi:10.4178/epih.e2022095)
Supplement: Supplementary Material 4. — t-test analysis of health-adjusted life expectancy with sex [file epih-44-e2022095-Supplementary-4.docx]

Supplementary Material 4. t-test analysis of health-adjusted life expectancy with sex

| Sex | Male | Female | *p* |
| --- | --- | --- | --- |
| Age | 55–70 | 55–70 |  |
| LE(x) (Total) | 21.38 (3.95) | 25.93 (4.40) |  |
| Physical activity |  |  | 0.8726 |
| HALE(x) (SD) | 8.62 (4.99) | 8.90 (5.06) |  |
| Participation in activities |  |  | 0.4391 |
| HALE (x) (SD) | 6.89 (3.70) | 7.91 (3.71) |  |
| Nutrition |  |  | 0.6726 |
| HALE (x) (SD) | 9.44 (5.91) | 8.63 (4.70) |  |

*Note:* HALE(x), health-adjusted life expectancy; SD, standard deviation; **p*<.05; ***p*<.001; ****p*<.0001
