# Supplementary material for: Health-adjusted life expectancy according to lifestyle classified by the Yonsei Lifestyle Profile-BREF
Source: Epidemiol Health. 2022 Oct 28;44:e2022095. doi: 10.4178/epih.e2022095 (PMC10396514; doi:10.4178/epih.e2022095)
Supplement: Supplementary Material 5. — ANOVA analysis of health-adjusted life expectancy with education attainment [file epih-44-e2022095-Supplementary-5.docx]

| Physical activity | Mean | S.D | F | *p* | post-hoc |
| --- | --- | --- | --- | --- | --- |
| Middle school or less | 2.13 | 1.82 | 5.70 | 0.0071^*^ | h>m^*^  c>m^*^ |
| High school | 10.38 | 6.89 |  |  |  |
| College degree | 8.17 | 4.59 |  |  |  |
| Participation in activities | Mean | S.D | F | *p* | post-hoc |
| Middle school or less | 2.83 | 1.87 | 4.48 | 0.0183^*^ | h>m^*^  c>m^*^ |
| High school | 7.64 | 4.28 |  |  |  |
| College degree | 7.06 | 3.52 |  |  |  |
| Nutrition | Mean | S.D | F | *p* | post-hoc |
| Middle school or less | 2.87 | 1.87 | 4.00 | 0.0269^*^ | h>m^*^  c>m^*^ |
| High school | 9.60 | 6.41 |  |  |  |
| College degree | 8.92 | 5.37 |  |  |  |

Supplementary Material 5. ANOVA analysis of health-adjusted life expectancy with education attainment

Note. m, middle school or less; h, high school; SD, standard deviation.

**p*<.05; ***p*<.001; ****p*<.0001
