# Supplementary material for: Health-adjusted life expectancy according to lifestyle classified by the Yonsei Lifestyle Profile-BREF
Source: Epidemiol Health. 2022 Oct 28;44:e2022095. doi: 10.4178/epih.e2022095 (PMC10396514; doi:10.4178/epih.e2022095)
Supplement: Supplementary Material 6. — ANOVA analysis of health-adjusted life expectancy with residential area [file epih-44-e2022095-Supplementary-6.docx]

Supplementary Material 6. ANOVA analysis of health-adjusted life expectancy with residential area.

| Physical activity | Mean | S.D | F | *p* | post-hoc |
| --- | --- | --- | --- | --- | --- |
| Urban | 8.44 | 4.69 | 1.25 | 0.2990 | - |
| Suburban | 9.24 | 5.69 |  |  |  |
| Rural | 12.06 | 5.60 |  |  |  |
| Participation in activities | Mean | S.D | F | *p* | post-hoc |
| Urban | 7.21 | 3.81 | 0.33 | 0.7204 | - |
| Suburban | 7.73 | 3.79 |  |  |  |
| Rural | 8.58 | 4.21 |  |  |  |
| Nutrition | Mean | S.D | F | *p* | post-hoc |
| Urban | 9.11 | 5.25 | 0.46 | 0.6368 | - |
| Suburban | 9.21 | 5.90 |  |  |  |
| Rural | 11.33 | 6.43 |  |  |  |

Note. SD, standard deviation.

**p*<.05; ***p*<.001; ****p*<.0001.
